# Association between vitamin D deficiency and risk of venous thromboembolism: a matched cohort study of 139,690 patients

**DOI:** 10.3389/fnut.2025.1639257

**Published:** 2025-09-17

**Authors:** Kuo-Chuan Hung, Li-Chen Chang, Chih-Wei Hsu, Jheng-Yan Wu, Chia-Hung Yu, Chun-Ning Ho, Ming Yew, I-Wen Chen

**Affiliations:** ^1^Department of Anesthesiology, Chi Mei Medical Center, Tainan, Taiwan; ^2^School of Medicine, College of Medicine, National Sun Yat-sen University, Kaohsiung, Taiwan; ^3^Department of Anesthesiology, E-Da Hospital, I-Shou University, Kaohsiung, Taiwan; ^4^Department of Psychiatry, Kaohsiung Chang Gung Memorial Hospital and Chang Gung University College of Medicine, Kaohsiung, Taiwan; ^5^Department of Nutrition, Chi Mei Medical Center, Tainan, Taiwan; ^6^Department of Anesthesiology, Chi Mei Medical Center, Liouying, Tainan, Taiwan

**Keywords:** vitamin D deficiency, venous thromboembolism, deep vein thrombosis, pulmonary embolism, cohort study, thrombosis prevention

## Abstract

**Background:**

Vitamin D deficiency (VDD) may contribute to venous thromboembolism (VTE) through effects on coagulation and endothelial function, but existing studies show inconsistent results. We investigated the association between VDD and VTE risk using a large matched cohort design.

**Methods:**

We conducted a retrospective matched cohort study using the TriNetX database, including patients aged ≥45 years with serum 25-hydroxyvitamin D (25(OH)D) measurements between 2010 and 2023. VDD was defined as serum 25(OH)D < 20 ng/mL, while controls had levels ≥30 ng/mL. After 1:1 propensity score matching, the final cohort comprised 69,845 patients in each group. Primary outcomes were deep vein thrombosis (DVT) and pulmonary embolism (PE) occurring 3–12 months after the index date. Secondary outcomes included all-cause mortality and intensive care unit (ICU) admission.

**Results:**

During one-year follow-up, VDD was significantly associated with increased risk of DVT (Hazard ratio [HR] 1.62, 95% confidence interval [CI]: 1.37–1.92; *p* < 0.001) and PE (HR 1.62, 95% CI: 1.34–1.96; *p* < 0.001) compared to controls. The association persisted over 2 years with modest attenuation (DVT: HR 1.49; PE: HR 1.61). A dose–response relationship was observed, with vitamin D insufficiency (20–30 ng/mL) showing intermediate risk levels (DVT: HR 1.36; PE: HR 1.43). VDD was also associated with higher mortality (HR 2.20, 95% CI: 1.99–2.43) and ICU admission risks (HR 1.47, 95% CI: 1.33–1.62). Subgroup analyses revealed consistent associations across demographic groups, with diabetes mellitus significantly modifying the DVT association.

**Conclusion:**

Vitamin D deficiency is independently associated with increased VTE risk in a dose-dependent manner, with effects extending to mortality and healthcare utilization. These findings support vitamin D optimization for VTE prevention, though randomized trials are needed to establish causality.

## 1 Introduction

Venous thromboembolism (VTE), which includes deep vein thrombosis (DVT) and pulmonary embolism (PE), imposes a substantial global health burden, affecting one to two individuals per 1,000 person-years in Europe and the United States (1, 2). Approximately 20% of individuals with VTE die within 1 year—often due to the underlying cause, and survivors frequently experience long-term complications, contributing to significant morbidity, healthcare burden, and mortality (1, 3–5). As efforts to prevent thrombotic events continue to evolve, a deeper understanding of multifactorial risk profiles is essential to identify modifiable factors and improve patient outcomes. Among the emerging risk factors, vitamin D deficiency (VDD) has garnered considerable attention owing to its potential mechanistic role in thrombosis and its widespread prevalence across diverse populations (6–8). VDD may contribute to a prothrombotic state through several mechanisms: impairment of endothelial integrity, upregulation of tissue factor expression, downregulation of anticoagulant proteins such as thrombomodulin, and dysregulation of the fibrinolytic system through altered plasminogen activator inhibitor-1 levels (6–8). These mechanistic insights have prompted numerous observational studies investigating the association between vitamin D status and VTE risk (9–13). Vitamin D also plays an important role in modulating the immune system. It enhances innate immunity and regulates adaptive immune responses by reducing pro-inflammatory cytokines and supporting immune homeostasis (14, 15). These immunomodulatory effects may reduce vascular inflammation and contribute to protection against venous thrombosis.

Our previous meta-analysis identified a significant association between low serum vitamin D levels and an elevated risk of VTE (16). However, the majority of the included studies were cross-sectional in nature, with only three longitudinal investigations (12, 13, 17) offering temporal evidence to support a potential causal relationship. A key limitation in the existing literature is the variability in the definitions of VDD, with cutoff values ranging from less than 20 ng/mL to less than 30 ng/mL, contributing to substantial heterogeneity in exposure classification (16). While meta-analysis (16) offers preliminary directional insight, substantial methodological variability across studies, including differences in study design, exposure assessment, outcome definition, and confounder adjustment, limits the robustness and generalizability of the pooled estimates. These limitations highlight the need for more methodologically rigorous research that minimizes design heterogeneity, adopts standardized definitions of vitamin D status, and uses appropriate statistical approaches to elucidate the relationship between vitamin D and VTE risk.

Based on established biological mechanisms and emerging clinical evidence, we hypothesized that VDD would be independently associated with increased VTE risk in a dose-dependent manner. The primary aim of this study was to conduct a comprehensive matched cohort analysis using a large-scale electronic health records database to quantify the association between VDD and VTE risk while controlling for confounding variables through propensity score matching.

## 2 Methods

### 2.1 Data sources and ethical statement

The TriNetX platform comprises electronic health records from over 150 million patients, with data contributed by healthcare organizations primarily in the United States, but also from Europe, Latin America, Asia-Pacific, and the Middle East and Africa regions (18). The database has been extensively utilized in published research to support a broad range of clinical studies (19–21). The TriNetX platform provides access to real-world clinical data, including demographics, diagnoses, procedures, medications, and laboratory results, enabling large-scale epidemiological research. The study protocol adhered to the principles outlined in the Declaration of Helsinki and was approved by the Institutional Review Board (IRB) of Chi Mei Medical Center (IRB number: 11310-E04). Given the use of de-identified data from an existing database, the requirement for individual informed consent was waived in accordance with the applicable regulations.

### 2.2 Study design and population

We conducted a matched cohort study including patients aged ≥ 45 years who had serum 25-hydroxyvitamin D [25(OH)D] measurements recorded between January 2010 and December 2023. Individuals aged over 45 years were selected for this study because the incidence of VTE and VDD increases substantially with age, particularly after midlife. Focusing on this age group improves the relevance and generalizability of our findings to populations at higher risk for both conditions. The index date was defined as the first documented measurement of either VDD [serum 25(OH)D < 20 ng/mL] for the VDD group or adequate vitamin D levels [serum 25(OH)D ≥ 30 ng/mL] for the control group. The control group consisted of patients with adequate vitamin D levels but were not healthy volunteers; rather, they were real-world patients without prior VTE. Both groups were well-matched for comorbidities through propensity score matching.

To ensure consistent vitamin D status and minimize misclassification bias, all patients were required to have a confirmatory measurement within their respective vitamin D category during a second measurement taken 3–12 months after the index date. Patients in the VDD group were excluded if any 25(OH)D measurement exceeded 20 ng/mL during the confirmation period. Similarly, for the control group, patients were excluded if any 25(OH)D measurement fell below 30 ng/mL during the same 3–12 month confirmation window. This bilateral exclusion criterion maintained group integrity by ensuring that the patients remained in their designated vitamin D status category throughout the confirmation period. The follow-up period was defined as 3–12 months after the index date. This washout period of 3 months was implemented to exclude VTE events that might have occurred due to acute conditions present at the time of vitamin D measurement, thereby focusing on the chronic effects of VDD on thrombotic risk.

### 2.3 Exclusion criteria

Patients were systematically excluded if they had any of the following conditions documented before the follow-up period (i.e., 3–12 months after the index date): a history of DVT or PE; HIV infection; organ transplantation; hemiplegia, hemiparesis, paraplegia, or quadriplegia; current hormone replacement therapy; or use of oral contraceptives.

Additionally, patients were excluded if they had any of the following conditions documented within 3 months prior to the follow-up period: pregnancy, lower extremity surgery, or cerebral infarction or hemorrhage. These exclusion criteria were designed to eliminate patients with established risk factors for VTE that could confound the association with VDD.

### 2.4 Data collection and propensity score matching

Patient characteristics extracted from the database included demographic variables (age, body mass index categories, sex, and race/ethnicity), comorbidities, laboratory values, and medications. Comorbidities assessed included essential hypertension, lipid disorders, diabetes mellitus, neoplasms, chronic kidney disease, ischemic heart disease, nicotine dependence, liver disease, heart failure, cerebrovascular disease, gout, alcohol-related disorders, COVID-19, rheumatoid arthritis, malnutrition, long-term steroid use, and systemic lupus erythematosus.

Laboratory parameters included hemoglobin A1c levels (≥7%), serum albumin (≥3.5 g/dL), hemoglobin levels (≥12 mg/dL), and estimated glomerular filtration rate (>60 mL/min/1.73m^2^). Medication use included ACE inhibitors, anticoagulants, platelet aggregation inhibitors, insulin and analogs, and angiotensin II receptor blockers.

To minimize confounding and selection bias, 1:1 propensity score matching was performed using a greedy matching algorithm without replacement. The propensity score was calculated using logistic regression, incorporating all collected baseline characteristics. Matching was performed with a caliper width of 0.1 standard deviations of the logit of the propensity score. The adequacy of matching was assessed using standardized mean differences, with values <0.1 considered indicative of adequate balance between groups.

### 2.5 Outcome definitions

The primary outcome was the development of VTE (i.e., DVT or PE) during the 3–12 month follow-up period after the index date. Secondary outcomes included all-cause mortality and intensive care unit admission during the same period. To assess the durability of the association and evaluate long-term effects, we expanded the follow-up period to 3–24 months after the index date in a separate analysis. This extended follow-up allows for the examination of whether the increased thrombotic risk associated with VDD persists over a longer time horizon, providing insight into the clinical significance and sustained impact of vitamin D status on cardiovascular outcomes.

### 2.6 Assessment of vitamin D insufficiency

To evaluate the dose–response relationship and assess whether milder degrees of VDD confer increased thrombotic risk, we conducted a separate analysis comparing patients with vitamin D insufficiency [serum 25(OH)D 20–30 ng/mL] to those with adequate vitamin D levels [serum 25(OH)D ≥ 30 ng/mL]. This analysis utilized the same inclusion and exclusion criteria, matching methodology, and follow-up periods (3–12 months) as the primary analysis, allowing for the assessment of a potential gradient effect of vitamin D status on VTE risk.

### 2.7 Subgroup analyses

Pre-specified subgroup analyses were performed to assess whether patient characteristics modified the association between VDD and VTE risk. Subgroups were defined by sex (male vs. female), age categories (>65 years vs. 45–65 years), diabetes mellitus status (present vs. absent), chronic kidney disease status (present vs. absent), and COVID-19 history (present vs. absent).

### 2.8 Statistical analysis

All statistical analyses were performed using the TriNetX analytics platform. Continuous variables are presented as means with standard deviations, while categorical variables are presented as counts with percentages. The association between VDD and study outcomes was assessed using Cox proportional hazards regression models, with the results reported as hazard ratios (HRs) with 95% confidence intervals (CIs). To account for multiple comparisons across the four primary outcome measures (DVT, PE, mortality, and ICU admission), we applied Bonferroni correction, with statistical significance defined as *p* < 0.0125 (0.05/4). In subgroup analyses, we set the threshold for statistical significance at *p* < 0.05 when testing for interactions. We determined whether subgroups differed significantly by examining the *p*-value for interaction, rather than simply comparing the CIs of estimates within each subgroup. To identify independent predictors of DVT and PE at 1-year follow-up, we performed multivariate Cox proportional hazards regression analyses including VDD and relevant baseline variables (e.g., age, sex, diabetes, chronic kidney disease, etc.). Results were presented as adjusted hazard ratios (aHRs) with 95% confidence intervals. All statistical tests were two-sided, and the analyses followed the intention-to-treat principle based on the vitamin D status at the index date.

## 3 Results

### 3.1 Patient selection and baseline characteristics

**Figure 1:**
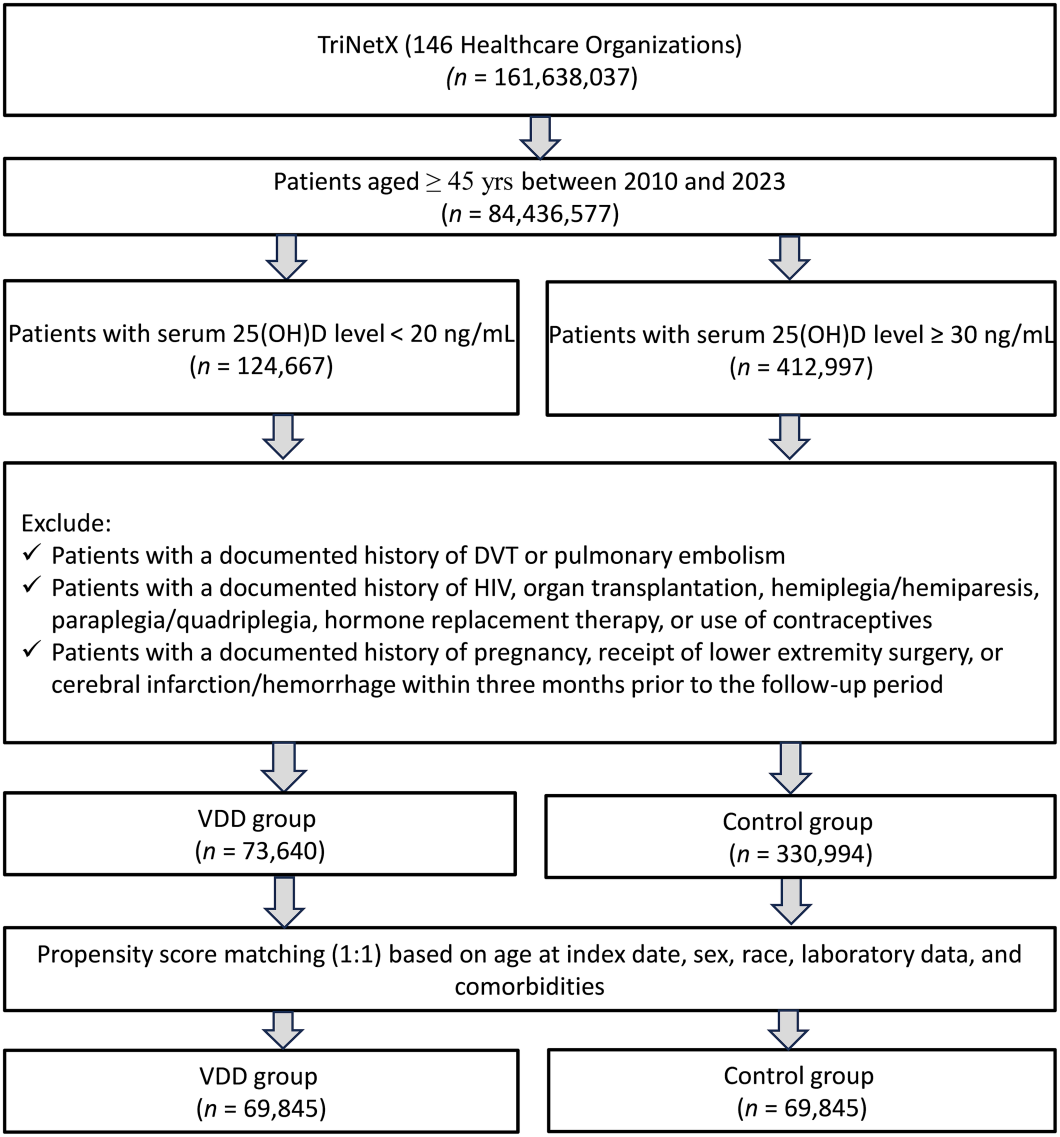
Patient selection from the TriNetX database.

A total of 161,638,037 patients from 146 healthcare organizations in the TriNetX database were screened. We included patients aged ≥45 years with available serum 25 (OH) D levels between 2010 and 2023. After applying these criteria, 124,667 patients with VDD and 412,997 control patients (serum 25(OH)D level ≥30 ng/mL) were identified. After applying the exclusion criteria, 73,640 patients with VDD and 330,994 patients with adequate vitamin D levels were included in the unmatched cohort. Following 1:1 propensity score matching, the final matched cohort comprised of 69,845 patients in each group, with a total of 139,690 patients (Figure 1).

Before matching, significant differences were observed between the VDD and control groups in terms of multiple baseline characteristics (Table 1). Patients with VDD were younger (60.3 ± 10.1 vs. 65.3 ± 10.0 years), had higher rates of obesity, and were less likely to be female (61.8% vs. 68.9%) compared to the control group. Racial distribution differed substantially, with VDD being more prevalent among Black or African American patients (21.7% vs. 8.3%) and less common among White patients (41.5% vs. 70.1%). The comorbidity profiles also differed significantly before matching. Patients with VDD have higher rates of diabetes mellitus, nicotine dependence, heart failure, and alcohol-related disorders. They also demonstrated poorer glycemic control and lower albumin levels. After propensity score matching, excellent balance was achieved across all measured variables (i.e., standardized mean differences <0.1 for all characteristics). The matched cohort had a mean age of 60.7 ± 10.1 years in the VDD group and 61.0 ± 9.9 years in the control group, with similar distributions of sex, race, comorbidities, laboratory values, and medications between groups.

**Table 1:** Baseline characteristics of patients before and after propensity score matching.

Variables	Before matching			After matching		
	VDD group (*n* = 73,640)	Control group (*n* = 330,994)	SMD^†^	VDD group (*n* = 69,845)	Control group (*n* = 69,845)	SMD^†^
Patient characteristics						
Age at index (years)	60.3 ± 10.1	65.3 ± 10.0	0.490	60.7 ± 10.1	61.0 ± 9.9	0.029
BMI 30–40 (kg/m^2^)	20,398 (27.7%)	76,527 (23.1%)	0.105	18,948 (27.1%)	19,922 (28.5%)	0.031
BMI 40–50 (kg/m^2^)	7,575 (10.3%)	20,760 (6.3%)	0.146	6,849 (9.8%)	7,050 (10.1%)	0.010
Female	45,492 (61.8%)	228,014 (68.9%)	0.150	44,011 (63.0%)	43,982 (63.0%)	0.001
White	30,533 (41.5%)	232,106 (70.1%)	0.603	30,311 (43.4%)	29,324 (42.0%)	0.029
Unknown Race	21,422 (29.1%)	45,837 (13.8%)	0.378	19,863 (28.4%)	20,638 (29.5%)	0.024
Black or African American	15,995 (21.7%)	27,485 (8.3%)	0.382	14,147 (20.3%)	14,312 (20.5%)	0.006
Asian	2,653 (3.6%)	16,374 (4.9%)	0.066	2,634 (3.8%)	2,678 (3.8%)	0.003
Other Race	2,454 (3.3%)	7,259 (2.2%)	0.070	2,346 (3.4%)	2,330 (3.3%)	0.001
Comorbidities						
Essential (primary) hypertension	32,766 (44.5%)	152,551 (46.1%)	0.032	30,543 (43.7%)	31,066 (44.5%)	0.015
Lipid disorders	28,855 (39.2%)	165,241 (49.9%)	0.217	27,412 (39.2%)	28,050 (40.2%)	0.019
Diabetes mellitus	20,297 (27.6%)	70,111 (21.2%)	0.149	18,301 (26.2%)	18,516 (26.5%)	0.007
Neoplasms	15,690 (21.3%)	86,104 (26.0%)	0.111	14,928 (21.4%)	14,607 (20.9%)	0.011
Chronic kidney disease (CKD)	10,057 (13.7%)	42,775 (12.9%)	0.022	9,050 (13.0%)	8,890 (12.7%)	0.007
Ischemic heart diseases	8,311 (11.3%)	35,858 (10.8%)	0.014	7,510 (10.8%)	7,257 (10.4%)	0.012
Nicotine dependence	7,942 (10.8%)	16,364 (4.9%)	0.218	6,494 (9.3%)	6,325 (9.1%)	0.008
Diseases of liver	5,611 (7.6%)	19,323 (5.8%)	0.071	4,997 (7.2%)	4,906 (7.0%)	0.005
Heart failure	5,586 (7.6%)	14,991 (4.5%)	0.128	4,689 (6.7%)	4,470 (6.4%)	0.013
Cerebrovascular diseases	3,467 (4.7%)	15,413 (4.7%)	0.002	3,148 (4.5%)	3,115 (4.5%)	0.002
Gout	2,445 (3.3%)	10,321 (3.1%)	0.011	2,261 (3.2%)	2,311 (3.3%)	0.004
Alcohol related disorders	2,642 (3.6%)	4,534 (1.4%)	0.143	2004 (2.9%)	1913 (2.7%)	0.008
COVID-19	1908 (2.6%)	9,847 (3.0%)	0.023	1805 (2.6%)	1,689 (2.4%)	0.011
Rheumatoid arthritis	1,642 (2.2%)	9,955 (3.0%)	0.049	1,597 (2.3%)	1,614 (2.3%)	0.002
Malnutrition	1931 (2.6%)	4,408 (1.3%)	0.093	1,563 (2.2%)	1,564 (2.2%)	0.000
Long term (current) use of steroids	1,394 (1.9%)	6,540 (2.0%)	0.006	1,322 (1.9%)	1,223 (1.8%)	0.011
Systemic lupus erythematosus (SLE)	601 (0.8%)	3,276 (1.0%)	0.018	584 (0.8%)	556 (0.8%)	0.004
Laboratory data						
HbA1c ≥ 7%	14,914 (20.3%)	37,664 (11.4%)	0.245	13,103 (18.8%)	13,163 (18.8%)	0.002
Albumin g/dL (≥3.5 g/dL)	55,233 (75.0%)	270,804 (81.8%)	0.166	52,771 (75.6%)	53,772 (77.0%)	0.034
Hemoglobin ≥ 12 mg/dL	59,225 (80.4%)	260,519 (78.7%)	0.043	56,094 (80.3%)	56,751 (81.3%)	0.024
eGFR>60 mL/min/1.73 m^2^	59,148 (80.3%)	257,113 (77.7%)	0.065	56,068 (80.3%)	56,482 (80.9%)	0.015
Medications						
ACE inhibitors	14,660 (19.9%)	49,838 (15.1%)	0.128	13,083 (18.7%)	13,238 (19.0%)	0.006
Anticoagulants	14,070 (19.1%)	49,998 (15.1%)	0.106	12,554 (18.0%)	12,407 (17.8%)	0.005
Platelet aggregation inhibitors	12,574 (17.1%)	50,823 (15.4%)	0.047	11,348 (16.2%)	10,978 (15.7%)	0.014
Insulins and analogues	11,870 (16.1%)	27,401 (8.3%)	0.241	10,097 (14.5%)	10,032 (14.4%)	0.003
Angiotensin II inhibitor	9,158 (12.4%)	47,339 (14.3%)	0.055	8,657 (12.4%)	8,840 (12.7%)	0.008

BMI, body mass index; SMD, standardized mean differences; ^†^SMD values <0.1 indicate adequate balance between groups; eGFR, estimated Glomerular Filtration Rate; ACE, Angiotensin-Converting Enzyme Inhibitors.

### 3.2 Outcomes

#### 3.2.1 Risk of VTE at 1-year follow-up

During the first year of follow-up, individuals with VDD exhibited a significantly higher risk of VTE than matched controls (Table 2). The incidence of DVT was 0.5% in the vitamin D-deficient cohort versus 0.3% among controls (HR 1.62, *p* < 0.001). PE was likewise more frequent in the deficiency group, occurring in 0.4% versus 0.2% of patients (HR 1.62, *p* < 0.001). Additionally, risks of mortality (HR 2.20, *p* < 0.001) and ICU admission (HR 1.47, *p* < 0.001) at 1 year were higher in those with VDD than in the control group. All reported associations remained statistically significant following Bonferroni correction (*p* < 0.0125).

**Table 2:** Association between vitamin D deficiency and 1-year outcomes.

Outcomes	VDD group (*n* = 69,845)	Control group (*n* = 69,845)	HR (95% CI)	Log-rank test: *p*-value^*^
	Events (%)	Events (%)		
DVT	344 (0.5%)	214 (0.3%)	1.62 (1.37–1.92)	< 0.001
Pulmonary embolism	278 (0.4%)	173 (0.2%)	1.62 (1.34–1.96)	< 0.001
Mortality	1,234 (1.8%)	565 (0.8%)	2.20 (1.99–2.43)	< 0.001
ICU admission	1,004 (1.4%)	689 (1.0%)	1.47 (1.33–1.62)	< 0.001

VDD, vitamin D deficiency; DVT, deep vein thrombosis; ICU, intensive care unit; HR, hazard ratio; CI: confidence interval. *P < 0.0125 was considered significance following Bonferroni correction.

#### 3.2.2 Risk of VTE at 2-year follow-up

The risk association persisted during the extended follow-up period (3–24 months post-index), which included 69,818 patients in each matched group (Table 3). Despite a modest attenuation in effect sizes over the extended follow-up period, VDD remained significantly linked to a higher risk of VTE. DVT was observed in 0.9% of the deficiency group versus 0.6% of the controls (HR 1.49, *p* < 0.001). Similarly, PE occurred more frequently in vitamin D-deficient patients (0.7%) than in those with adequate levels (0.4%; HR 1.61, *p* < 0.001). By the two-year mark, excess risks extended to secondary outcomes as well. The VDD group experienced higher risks of all-cause mortality (HR 1.88, *p* < 0.001) and ICU admission (HR 1.42, *p* < 0.001) relative to controls.

**Table 3:** Association between vitamin D deficiency and 2-year outcomes.

Outcomes	VDD group (*n* = 69,818)	Control group (*n* = 69,818)	HR (95% CI)	*p*-value*
	Events (%)	Events (%)		
DVT	617 (0.9%)	416 (0.6%)	1.49 (1.31–1.68)	< 0.001
Pulmonary embolism	480 (0.7%)	298 (0.4%)	1.61 (1.40–1.86)	< 0.001
Mortality	2,363 (3.4%)	1,260 (1.8%)	1.88 (1.75–2.01)	< 0.001
ICU admission	1874 (2.7%)	1,319 (1.9%)	1.42 (1.33–1.53)	< 0.001

VDD, vitamin D deficiency; DVT, deep vein thrombosis; ICU, intensive care unit; HR, hazard ratio; CI, confidence interval. *P < 0.0125 was considered significance following Bonferroni correction.

### 3.3 Association between vitamin D insufficiency and VTE risk

To compare vitamin D insufficiency (20–30 ng/mL) with adequate levels (≥30 ng/mL), 102,326 matched pairs were analyzed (Table 4). The results demonstrated a gradient in risk, as patients with vitamin D insufficiency experienced intermediate rates of adverse outcomes compared to those with deficiency or sufficiency. After 1 year, vitamin D insufficiency was associated with an increased risk of DVT (HR 1.36, *p* < 0.001) and PE (HR 1.43, *p* < 0.001). These individuals also had higher risks of mortality (HR 1.25, *p* < 0.001) and ICU admission (HR 1.31, *p* < 0.001). The two-year analysis confirmed the persistence of these associations. DVT risk remained elevated (HR 1.23, *p* < 0.001), as did PE risk (HR 1.30, *p* < 0.001), mortality (HR 1.18, *p* < 0.001), and ICU admissions (HR 1.22, *p* < 0.001).

**Table 4:** Association between vitamin D insufficiency and outcomes.

Outcomes	1-year (*n* = 102,326 for each group)		2-year (*n* = 102,326 for each group)	
	HR (95% CI)	*p*-values	HR (95% CI)	*p*-values*
DVT	1.36 (1.13–1.58)	< 0.001	1.23 (1.09–1.38)	< 0.001
Pulmonary embolism	1.43 (1.19–1.71)	< 0.001	1.30 (1.14–1.48)	< 0.001
Mortality	1.25 (1.13–1.38)	< 0.001	1.18 (1.10–1.26)	< 0.001
ICU admission	1.31 (1.18–1.45)	< 0.001	1.22 (1.14–1.31)	< 0.001

Vitamin D insufficiency was defined as a serum 25(OH)D level between 20 and 30 ng/mL; DVT, deep vein thrombosis; ICU, intensive care unit; HR, hazard ratio; CI, confidence interval. *p < 0.013 was considered significance following Bonferroni correction.

### 3.4 Subgroup analyses

Subgroup analyses were conducted to explore potential effect modifiers of the association between VDD and the risk of DVT at 1 year (Table 5). The association remained consistent across sexes, with a similar effect size observed in males (HR 1.57) and females (HR 1.53; p-interaction = 0.887). When stratified by age, the association was stronger in individuals aged 45–65 years (HR 1.80) compared to those over 65 years (HR 1.40), though the interaction was not statistically significant (*p*-interaction = 0.207). Diabetes mellitus significantly modified the association (*p*-interaction = 0.022), with a more pronounced effect in patients without diabetes (HR 1.90) than in those with diabetes (HR 1.26). A similar trend was observed for chronic kidney disease (CKD), with a higher risk among those without CKD (HR 1.67) versus those with CKD (HR 1.21), although the interaction did not reach statistical significance (*p*-interaction = 0.090). History of COVID-19 infection did not significantly influence the association (*p*-interaction = 0.555), with comparable hazard ratios in patients with (HR 1.40) and without (HR 1.61) prior infection.

**Table 5:** Subgroup analysis of association between vitamin D deficiency and risk of deep vein thrombosis at 1-year follow-up.

Subgroup analysis	HR (% CI)	*P*-value	*P* for interaction
Sex			0.887
Male	1.57 (1.2–2.05)	<0.001	
Female	1.53 (1.22–1.92)	<0.001	
Age			0.207
>65 years	1.4 (1.14–1.7)	0.001	
45–65 years	1.8 (1.33–2.44)	<0.001	
Diabetes Mellitus			0.022
Yes	1.26 (0.95–1.67)	0.109	
No	1.9 (1.53–2.36)	<0.001	
Chronic kidney disease			0.090
Yes	1.21 (0.86–1.7)	0.269	
No	1.67 (1.38–2.03)	<0.001	
COVID-19			0.555
Yes	1.40 (0.9–2.16)	0.13	
No	1.61 (1.34–1.94)	<0.001	

HR, hazard ratio; CI, confidence interval.

Regarding PE, the association with VDD remained consistent across subgroups, with no significant effect modification. Notably, patients with a history of COVID-19 displayed a higher risk (HR 2.72, *p* < 0.001) compared to those without COVID-19 (HR 1.68, *p* < 0.001), although this difference did not reach statistical significance (*p*-interaction = 0.198) (Table 6).

**Table 6:** Subgroup analysis of association between vitamin D deficiency and risk of pulmonary embolism at 1-year follow-up.

Subgroup analysis	HR (% CI)	*P*-value	*P* for interaction
Sex			0.652
Male	1.62 (1.18–2.23)	0.003	
Female	1.78 (1.38–2.29)	<0.001	
Age			0.657
>65 years	1.69 (1.35–2.13)	<0.001	
45–65 years	1.54 (1.1–2.17)	0.011	
Diabetes Mellitus			0.958
Yes	1.76 (1.25–2.47)	<0.001	
No	1.78 (1.4–2.27)	<0.001	
Chronic kidney disease			0.584
Yes	1.68 (1.1–2.58)	0.016	
No	1.92 (1.53–2.4)	<0.001	
COVID-19			0.198
Yes	2.72 (1.58–4.67)	<0.001	
No	1.68 (1.36–2.07)	<0.001	

HR, hazard ratio; CI, confidence interval.

### 3.5 Multivariate predictors of DVT and PE

In multivariate Cox regression models at 1-year follow-up, VDD remained independently associated with an increased risk of DVT (aHR 1.71, *p* < 0.001) (Table 7) and PE (aHR 1.97, *p* < 0.001) (Table 8), after adjusting for age, sex, diabetes, chronic kidney disease, and other relevant covariates.

**Table 7:** Multivariate predictors of deep vein thrombosis at 1-year follow-up.

Variable	Hazard Ratio (95% CI)	*P*-value
VDD vs. control groups	1.71 (1.51–1.93)	<0.001
Male	1.22 (1.09–1.36)	0.001
Age at Index	1.02 (1.02–1.03)	<0.001
Essential (primary) hypertension	1.19 (1.05–1.34)	0.006
Overweight and obesity	1.24 (1.08–1.42)	0.003
Nicotine dependence	1.24 (1.03–1.50)	0.023
Ischemic heart diseases	1.23 (1.05–1.43)	0.011
Chronic kidney disease (CKD)	1.41 (1.21–1.63)	<0.001
Chronic obstructive pulmonary disease	1.48 (1.24–1.78)	<0.001
Malnutrition	3.89 (3.09–4.90)	<0.001
Heart failure	1.40 (1.16–1.70)	0.001
Diabetes mellitus	1.15 (1.01–1.31)	0.038

VDD, vitamin D deficiency.

**Table 8:** Multivariate predictors of pulmonary embolism at 1-year follow-up.

Variable	Hazard Ratio (95% CI)	*P*-value
VDD vs. control groups	1.97 (1.72–2.26)	<0.001
Male	1.19 (1.05–1.35)	0.008
Age at Index	1.02 (1.02–1.03)	<0.001
Essential (primary) hypertension	1.16 (1.01–1.33)	0.035
Overweight and obesity	1.46 (1.25–1.71)	<0.001
Nicotine dependence	1.08 (0.86–1.35)	0.529
Ischemic heart diseases	1.01 (0.83–1.22)	0.946
Chronic kidney disease (CKD)	1.23 (1.03–1.46)	0.024
Chronic obstructive pulmonary disease	1.72 (1.41–2.12)	<0.001
Malnutrition	3.23 (2.43–4.30)	<0.001
Heart failure	1.89 (1.52–2.34)	<0.001
Diabetes mellitus	0.91 (0.77–1.06)	0.211

VDD, vitamin D deficiency.

## 4 Discussion

This large-scale matched cohort study of 139,690 patients demonstrated a significant association between VDD and an increased risk of VTE. Our findings reveal that individuals with VDD face substantially higher risks of both DVT and PE, with HRs of 1.62 for each condition at one-year follow-up. This association persisted over extended follow-up periods and demonstrated a dose-dependent relationship, with vitamin D insufficiency showing intermediate risk levels between deficiency and sufficiency. Additionally, VDD was associated with increased mortality and intensive care unit admission rates, suggesting broader clinical implications beyond thrombotic events.

The existing literature on VDD and VTE has been characterized by significant methodological limitations and inconsistent findings (16). Most previous investigations have employed cross-sectional designs focusing on specific high-risk populations, including patients undergoing lower-limb surgery, stroke patients, brain injury cases, and spinal cord injury populations (9, 11, 22–24). While these studies have provided valuable insights into vitamin D status in thrombosis-prone populations, their cross-sectional nature limits their ability to establish temporal relationships and causal inference. Only three large longitudinal cohort studies have examined this association in the general population, all published between 2013 and 2014 (12, 13, 17). Notably, the findings from these studies were inconsistent, with two of the three studies failing to demonstrate a significant association between vitamin D status and VTE risk (12, 13). Furthermore, these investigations evaluated long-term associations with follow-up periods ranging from 10.7 to 30 years, which may not provide clinically relevant information for short-term risk stratification and prevention strategies. The extended follow-up periods in these studies may have diluted the observable effects due to changes in vitamin D status over time, lifestyle modifications, and the development of competing risks that could mask the association.

Our findings align with and strengthen the evidence from previous meta-analyses investigating the relationship between vitamin D status and VTE risk (16, 25). Our prior meta-analysis demonstrated a negative relationship between vitamin D levels and VTE risk, with pooled odds ratios of 1.74 (95% CI: 1.37–2.20) for low vitamin D status and hazard ratios of 1.25 (95% CI: 1.07–1.46) from longitudinal studies. Our study corroborates these findings with similar effect sizes. However, our results contrast with those of some earlier longitudinal cohort studies. Two of the three large general population cohort studies from to 2013–2014 found no significant association between vitamin D levels and VTE risk. This discrepancy may be attributed to several factors, including differences in study populations, varying definitions of VDD, extended follow-up periods that may have obscured short-term associations, and potential confounding variables that were not adequately controlled for in earlier studies. The consistency of our findings across both DVT and PE suggests that VDD affects the entire spectrum of VTE rather than being specific to particular manifestations. This supports the biological plausibility of the role of vitamin D in thrombosis through its effects on multiple pathways involved in coagulation, endothelial function, and inflammation.

Our investigation makes several novel contributions to the literature. First, this is the largest matched cohort study to date examining the association between VDD and VTE risk, with 139,690 participants providing substantial statistical power to detect clinically meaningful associations. Second, our study design addresses critical limitations of previous research by employing a shorter, more clinically relevant follow-up period (3–12 months) that captures the acute effects of VDD on thrombotic risk while minimizing confounding from temporal changes in vitamin D status. Third, our rigorous propensity score matching methodology ensured balanced comparison groups across multiple demographic, clinical, and laboratory parameters, thereby reducing selection bias and confounding factors that may have influenced previous observational studies. Fourth, we provide evidence of a dose–response relationship by separately analyzing VDD (<20 ng/mL) and insufficiency (20–30 ng/mL), demonstrating graduated risk levels that support a causal relationship. Finally, we provide strong population-based evidence from a large, diverse group of patients, moving beyond the specialized patient groups (patients undergoing lower-limb surgery, stroke patients, brain injury cases, and spinal cord injury populations) that most previous studies have focused on (9, 11, 22–24).

The observed association between VDD and increased mortality risk (HR 2.20) represents a particularly concerning finding that extends beyond primary thrombotic outcomes. This association may reflect multiple pathways through which VDD influences clinical outcomes. The increased mortality could be directly related to the higher incidence of fatal PE in vitamin D-deficient patients (26). However, mortality risk likely encompasses broader mechanisms beyond thrombotic events. VDD has been associated with immune dysfunction, increased susceptibility to infections, cardiovascular disease, and overall frailty, all of which could contribute to an increased mortality risk (27–29). The observed magnitude of the association with mortality (HR 2.20) surpasses that observed for thrombotic events alone, suggesting that VDD may reflect broader impairments in overall health and physiological reserve, or alternatively, that it contributes directly to mortality through multiple overlapping biological pathways. The persistence of elevated mortality risk over the two-year follow-up period (HR 1.88) indicates that VDD represents a sustained risk factor rather than merely an acute marker of poor health status.

Our subgroup analyses provided important insights into the consistency and potential modifiers of the vitamin D-VTE association. For DVT, the association remained robust across sex and age groups, suggesting that VDD represents a universal risk factor, regardless of these demographic characteristics. However, the significant interaction with diabetes mellitus (*p*-interaction = 0.022) revealed important clinical nuances, with stronger associations observed in patients without diabetes. This interaction may reflect a complex relationship between vitamin D status, glucose metabolism, and coagulation. Patients with diabetes may have multiple competing risk factors for thrombosis, potentially diluting the observable effect of VDD (30, 31). Alternatively, diabetes-related medications or metabolic management strategies may modify the relationship between vitamin D status and thrombotic risk. For PE, the absence of significant effect modification across most subgroups supports the generalizability of our findings. The notably higher risk observed in patients with COVID-19 history (HR 2.72) than in those without (HR 1.68), while not statistically significant for interaction, may reflect the synergistic effects of COVID-19-related hypercoagulability and VDD-associated prothrombotic states.

Several limitations must be acknowledged when interpreting our findings. First, as an observational study, we cannot definitively establish causality despite the temporal sequence and dose–response relationship observed. Residual confounding from unmeasured variables, including genetic factors, dietary patterns, physical activity levels, and seasonal variations in vitamin D synthesis (32, 33), may influence our results. Second, our study relied on electronic health record data, which may be subject to coding inaccuracies, missing data, and selection bias toward patients who seek healthcare more frequently. The requirement for vitamin D testing may introduce a selection bias toward patients with suspected deficiency or those receiving more comprehensive medical evaluation. Third, we did not account for seasonal variations in vitamin D levels, which could influence both exposure classification and outcome risk. Vitamin D status can fluctuate significantly throughout the year, and our confirmation period of 3–12 months may not fully capture these temporal variations. Fourth, we lacked detailed information on vitamin D supplementation use, sun exposure patterns, dietary vitamin D intake, and other factors that could modify the vitamin D status over time. Additionally, we could not assess the potential confounding effects of certain medications that may influence both vitamin D metabolism and the risk of thrombosis. Fifth, our study population was derived from healthcare systems that may not be representative of the broader population, potentially limiting the generalizability of our findings to different demographic groups, geographic regions, or healthcare settings.

## 5 Conclusion

This large-scale matched cohort study provides evidence that VDD is independently associated with an increased risk of VTE, with a dose-dependent relationship and persistent effects over time. This association extends beyond thrombotic events to include increased mortality and intensive care utilization, suggesting broad clinical implications. Our findings support the biological plausibility of the antithrombotic properties of vitamin D and provide epidemiological evidence that may inform clinical practice guidelines for VTE prevention. Given the modifiable nature of VDD and the substantial public health burden of VTE, these findings warrant serious consideration for prevention strategies. However, randomized controlled trials of vitamin D supplementation are needed to establish causality and determine optimal dosing strategies for VTE prevention. Future research should also investigate the mechanisms underlying the association between vitamin D-VTE association and identify patient populations that might benefit most from vitamin D optimization as part of comprehensive thrombosis prevention strategies.

## Data Availability

The raw data supporting the conclusions of this article will be made available by the authors, without undue reservation.
